# Expression of estrogen receptor beta isoforms in pancreatic adenocarcinoma

**DOI:** 10.18632/oncotarget.26503

**Published:** 2018-12-28

**Authors:** Mamoun Younes, Charles J. Ly, Kanchan Singh, Atilla Ertan, Pamela S. Younes, Jennifer M. Bailey

**Affiliations:** ^1^ Departments of Pathology and Laboratory Medicine, University of Texas Health Science Center at Houston McGovern Medical School, Houston, TX, USA; ^2^ Department of Medicine, Section of Gastroenterology, Hepatology and Nutrition, University of Texas Health Science Center at Houston McGovern Medical School, Houston, TX, USA

**Keywords:** estrogen receptor, proliferative activity, quantitative biomarker analysis, image analysis, pancreas

## Abstract

Limited studies have shown that some patients with pancreatic adenocarcinoma (PAC) may benefit from treatment with tamoxifen. PAC has been shown to be largely negative for estrogen receptor alpha (ER-alpha). The aim of this pilot study was to investigate ER-beta expression in human PAC. Sections of tissue microarray with 18 evaluable cases of human PAC were stained by immunohistochemistry (IHC) for ER-beta1, ER-beta2, ER-beta5, and Cyclin A. The levels of ER-beta isoform expression and the S-phase fraction (SPF) were determined using quantitative digital image analysis.

Higher mean and median ER-beta2 levels correlated with male sex (*p* = 0.057 and *p* = 0.035, respectively), older age (*p* = 0.005 and *p* = 0.006, respectively), and lower pT stage (*p* = 0.008 and *p* = 0.009). Mean and median ER-beta5 levels correlated negatively with SPF (*p* = 0.021 and *p* = 0.047, respectively). Mean ER-beta1 expression did not correlate with any of the above mentioned clinicopathologic factors. The findings in this pilot study, although should be considered preliminary, suggest that some ER-beta isoforms may play a role in the biology of PAC. Additional larger studies are needed to confirm our findings, and to determine whether ER-beta may be considered for future targeted therapy.

## INTRODUCTION

It has been estimated that 53,670 individuals will be diagnosed with pancreatic adenocarcinoma (PAC) and 43,090 will die of it in the United States in 2017 [1]. The 5-year survival for PAC is 8%, which is thought to be largely due to the fact that the majority of PAC are diagnosed at and advanced stage [1]. There has been no significant progress in the treatment of PAC despite increasing knowledge of the molecular biology and pathogenesis of this tumor [2]. *In vitro* studies showed that PAC cell lines express estrogen receptor beta (ER-β, and that growth of PAC cell lines that express estrogen receptors can be regulated by estrogens and antiestrogens [3]. The aim of this pilot study was to investigate ER-β protein expression in human PAC using immunohistochemistry (IHC).

## RESULTS

**Figure 1:**
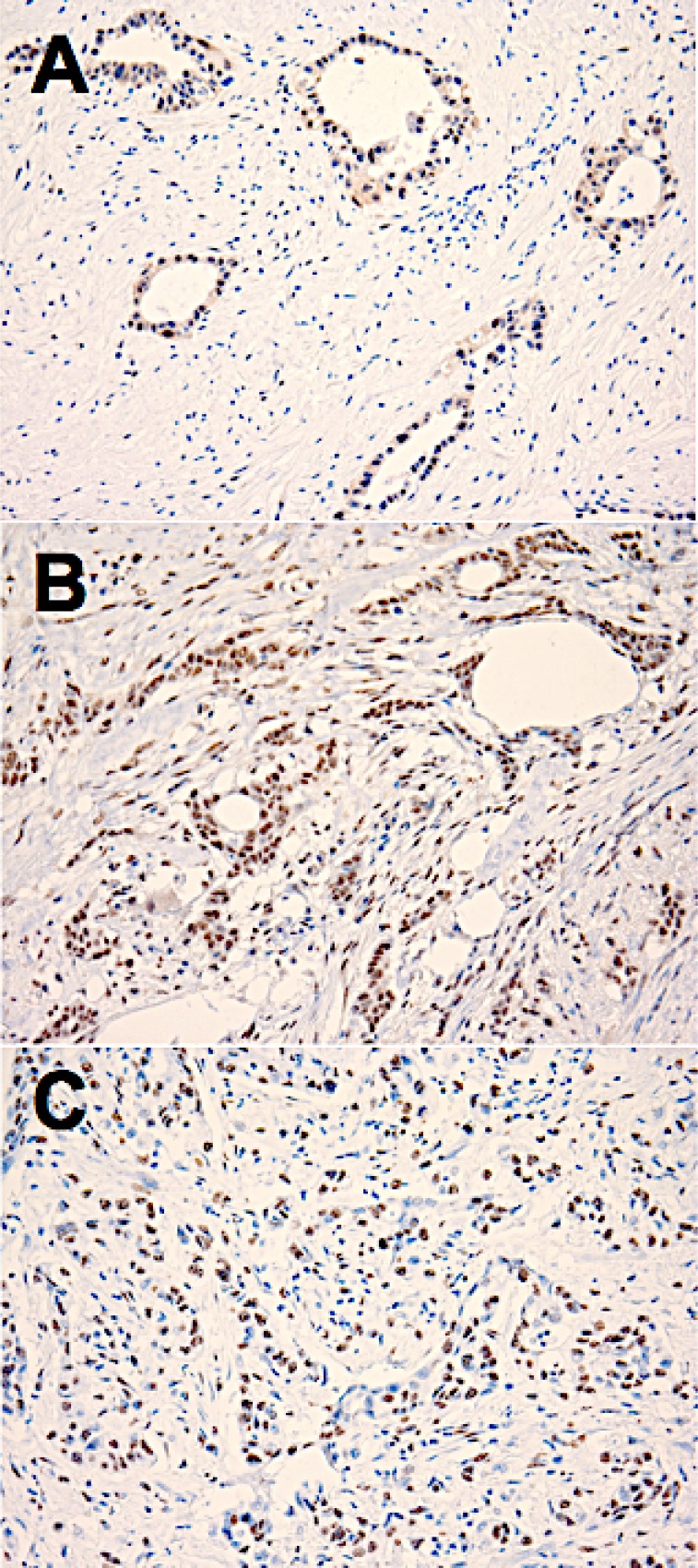
Examples of positive immunohistochemical staining for estrogen receptor (ER)-beta1 (**A**), ER-beta2 (**B**) and ER-beta5 (**C**) in sections of human pancreatic adenocarcinoma tissue. 20× microscope objective.

**Figure 2:**
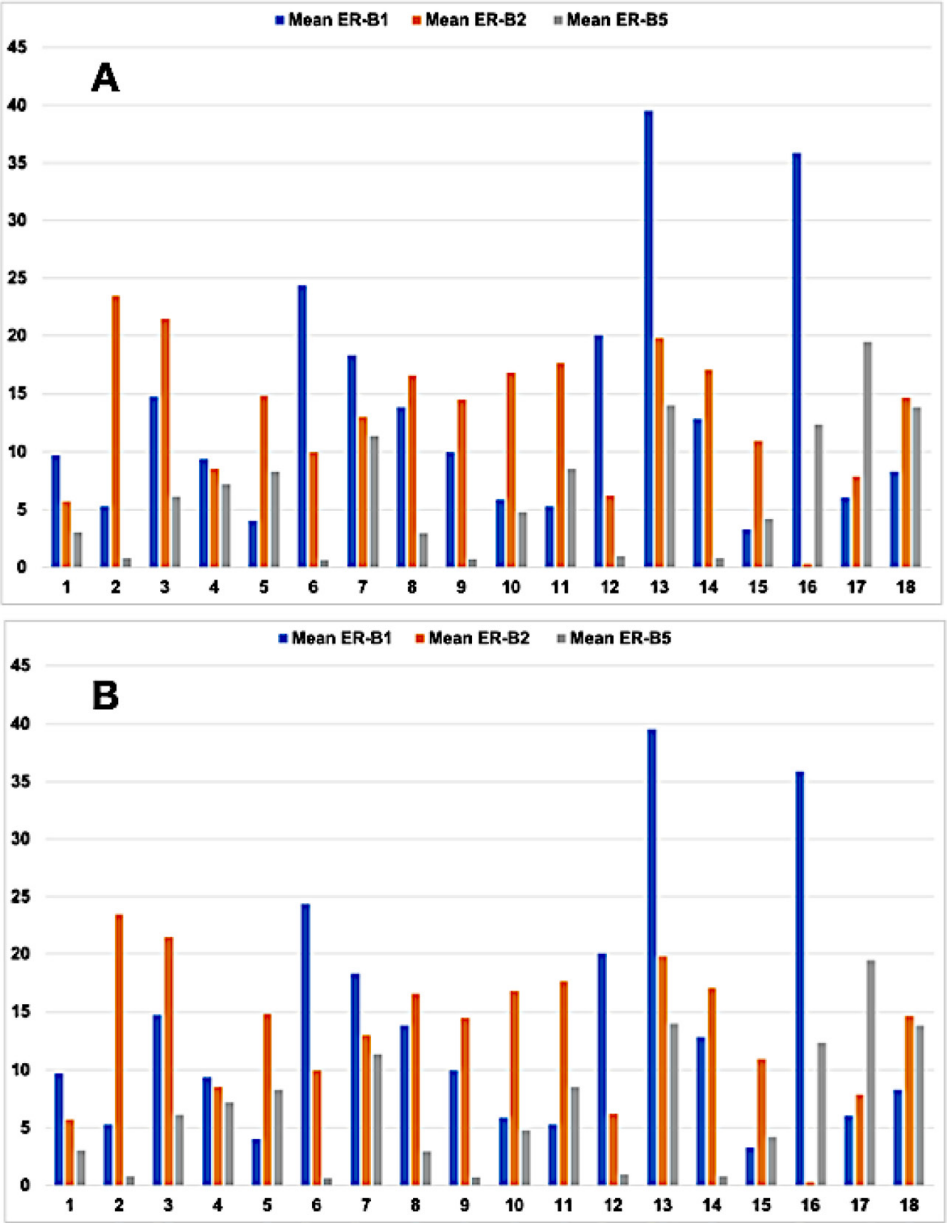
Bar graph showing the mean (**A**) and median (**B**) levels of expression of estrogen receptor beta (ER-B) in 18 cases of human pancreatic adenocarcinoma (PAC). ER-B1 (blue), ER-B2 (orange) and ER-B5 (gray). X axis: case number. Y axis: level of ER-beta isoform expression in OTMIAS units (OU).

**Figure 3:**
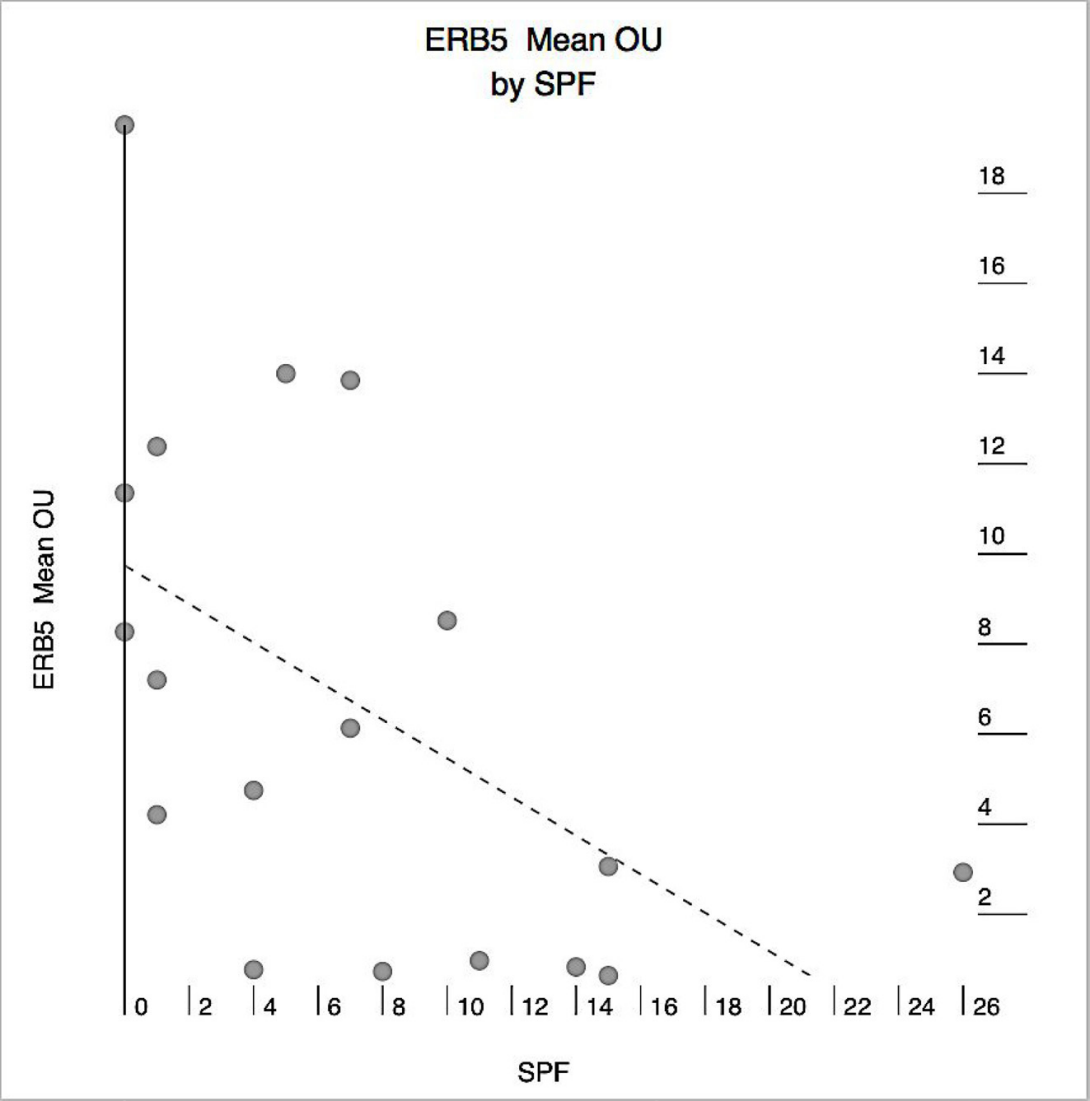
Correlation between mean estrogen receptor-beta5 (ERB5) expression in human pancreatic adenocarcinoma (PAC) and the tumor proliferative activity as measured by the S-phase fraction (SPF) X axis: SPF. Y axis: mean ERB5 expression in OTMIAS units (OU). There is significant negative correlation (Pearson correlation, *p* = 0.021).

Tumor characteristics of the 18 cases of PAC used in this study are shown in Table 1. All three ER-β isoforms were expressed in PAC (Figure 1), although the levels of expression of each isoform varied between tumors (Figure 2). Because of the small sample size studied from each tumor (each core is 2 mm), heterogeneity of ER-β expression within each individual adenocarcinoma cannot be determined from this study. Higher mean and median ER-β2 levels correlated with male sex (*p* = 0.057 and *p* = 0.035, respectively), older age (*p* = 0.005 and *p* = 0.006, respectively), and lower pT stage (*p* = 0.008 and *p* = 0.009), but not with pathologic lymph node stage (pN), or the tumor S-phase fraction (SPF). Mean and median ER-β5 levels correlated negatively with SPF (*p* = 0.021 and *p* = 0.047, respectively, Figure 3), but not with sex, age, pT or pN. Mean ER-β1 expression did not correlate with any of the above mentioned clinicopathologic factors.

**Table 1:** Patient and tumor characteristics for the 18 cases of pancreatic adenocarcinoma entered in the study

Case number	Age	Sex	Grade	TNM Stage
1	44	Female	1	T3N1M0
2	64	Female	2	T2N0M0
3	56	Male	1	T2N0M0
4	49	Female	1	T3N0M0
5	53	Female	2	T2N0M0
6	49	Female	2	T3N1M1
7	52	Female	2	T4N1M1
8	42	Female	2	T3N0M0
9	58	Male	3	T3N0M0
10	60	Female	3	T3N1M0
11	53	Female	2	T2N0M0
12	69	Male	1	T3N1M0
13	40	Female	2	T3N0M0
14	61	Male	3	T2N0M0
15	76	Female	3	T3N1M0
16	53	Female	2	T3N1M0
17	51	Female	3	T3N0M0
18	42	Female	3	T3N0M0

## DISCUSSION

Limited trials in which tamoxifen efficacy was tested in patients with pancreatic adenocarcinoma (PAC) yielded contradicting results [4–7]. In patients with breast cancer, the effect of tamoxifen on survival is related to the expression of estrogen receptor (ER) in breast cancer cells, and the higher the expression the greater the benefit of treatment with tamoxifen [8]. The contradicting results on the benefit of adjuvant tamoxifen in PAC is likely because patients were not stratified by ER status. In the past few decades, ER status in cancer tissue was determined using immunohistochemistry (IHC) on sections of formalin-fixed and paraffin-embedded tissues., and PAC was believed to be ER-negative [9, 10]. In these studies, IHC for ER was done using antibodies only the traditional ER, now referred to as ER-alpha.

A newer class of estrogen receptors has been identified and called ER-β [11–14]. This receptor has several isoforms, and appears to have a wider tissue distribution at the mRNA level [15]. The traditional ER is now called estrogen receptor alpha (ER-alpha). Utilizing IHC, ER-β has been identified in several human cancer types that were traditionally known to be ER-negative based on previous assays using ER-alpha.-specific antibodies, including carcinomas of the colon, esophagus, stomach, lung, and prostate [16–21]. We have previously shown the ER-β expression as detected by IHC is a significant predictor of survival in breast cancer patients treated with tamoxifen independent of ER-alpha expression [22–25].

Although ER-β mRNA has been previously detected in human PAC [26], and ER-β has been shown to play a significant role in estrogen-induced growth stimulation of pancreatic cancer cell lines [3], the expression of ER-β proteins in human PAC tissue is still largely unknown. The findings in our current study show that ER-β1, ER-β2, and ER-β5 are expressed in PAC at different levels. More importantly, we found that higher expression levels of two of these isoforms, ER-β2 and ER-β5, correlate significantly with various patient and tumor characteristics indicating that they may play an important role in the biology of PAC. Of particular interest was the finding of negative correlation between mean and median ER-β5 and SPF, which raise the possibility that ER-β5 may suppress PAC proliferation. The results of this small pilot study are preliminary, but we hope will stimulate larger studies to evaluate the role of ER-β isoforms as potential treatment targets in PAC, taking into consideration the levels of expression of each isoform in the target tumor, patient age, gender, use of exogenous steroidal hormones, and the pancreatic cancer subtypes.

## MATERIALS AND METHODS

Sections of tissue microarray containing 18 formalin fixed and paraffin embedded human PAC PAC (Cat # Z7020090 BioChain, https://www.biochain.com/) were stained by immunohistochemistry (IHC) using monoclonal antibodies to ER-β 1 (clone PPG5/10), ER-β 2 (clone 57/3) and ER-β 5 (clone 5/25) (all from abd Serotec, now Bio-Rad), and for Cyclin A (Clone 6E6, Abcam). Briefly, following steam heat antigen retrieval in low pH buffer (ER-β2) or high pH buffer (ER-β1 and ER-β5), or in pressure cooker in low pH buffer (cyclin A), sections were incubated for 30 minutes at room temperature with 1:10 dilution of ER-β1, 1:20 dilution of ER-β2 antibody, 1:40 dilution of ER-β5 antibody, or 1:200 dilution of the cyclin-A antibody. Immunoperoxidase staining was carried out utilizing a Dako automated immunostainer (Agilent Technologies) and a universal peroxidase detection kit (Dako). Appropriate positive and negative control cell lines were used. The levels of ER-β isoform expression in tumor cells and the S-phase fraction (SPF) were determined using a quantitative digital image analysis solution (OTMIAS, Olive Tree Media, LLC, https://www.biochain.com/) [27]; expression levels are given in OTMIAS Units (OU) which are based on the relative value of expression in each tumor cell compared to similarly processed positive and negative cell lines standards. All evaluations were done in a blinded fashion. Statistical analysis was performed utilizing Wizard statistical software for Apple Mac OS (Evan Miller, available from the App Store). *p* & 0.05 is considered statistically significant.
